# Dynamics of Sows’ Activity Housed in Farrowing Pens with Possibility of Temporary Crating might Indicate the Time When Sows Should be Confined in a Crate before the Onset of Farrowing

**DOI:** 10.3390/ani10010006

**Published:** 2019-12-18

**Authors:** Maciej Oczak, Kristina Maschat, Johannes Baumgartner

**Affiliations:** 1 Institute of Animal Welfare Science, The University of Veterinary Medicine Vienna (Vetmeduni Vienna), Veterinärplatz 1, 1210 Vienna, Austria; Kristina.Maschat@vetmeduni.ac.at (K.M.); Johannes.Baumgartner@vetmeduni.ac.at (J.B.); 2 Precision Livestock Farming Hub, The University of Veterinary Medicine Vienna (Vetmeduni Vienna), Veterinärplatz 1, 1210 Vienna, Austria; 3 FFoQSI GmbH, Technopark 1C, A-3430 Tulln, Austria

**Keywords:** farrowing prediction, accelerometer, precision livestock farming, nest-building, farrowing crate

## Abstract

**Simple Summary:**

In natural conditions, before the onset of farrowing, sows build a nest mainly to protect piglets from adverse weather conditions and predators. In modern commercial farms nest-building behaviour is limited, because, during the period of nest building, sows are confined in a farrowing crate. This has a negative effect on sow welfare. Recently, the concept of temporary crating has been introduced to achieve a compromise between the needs of the sow and her piglets. According to this concept, sows could be kept out of the crate at the time of prenatal nest-building behaviour and after the critical period of piglets’ life. In this study, we developed a method to monitor sows’ behaviour on the basis of accelerometer data. The developed monitoring method provides the following two types of alarms: A “first-stage” alarm that indicates the beginning of nest-building behaviour, with a median of 8 h 51 min before the onset of farrowing and a “second-stage” alarm that indicates the end of nest-building behaviour with a median of 2 h 3 min before the onset of farrowing. On the basis of these two types of alarms a farmer can make a decision when to provide adequate nest-building material and when to confine a sow in a crate for protection of the piglets’ welfare.

**Abstract:**

One way to reduce the negative impact of farrowing crates on sow welfare is to limit confinement of sows from the onset of farrowing until the end of the critical period of piglets’ life a few days after farrowing. In order to provide an indication of the time when sows should be confined in crates, ear tag-based acceleration data was modeled to provide the following two types of alarms: A “first-stage” alarm that indicates the beginning of nest-building behaviour, and a “second-stage” alarm that indicates the ending of the nest-building behaviour. In total, 53 sows were included in the experiment. Each sow had an ear tag with an accelerometer sensor mounted on the ear. Acceleration data were modeled with the Kalman filtering and fixed interval smoothing (KALMSMO) algorithm. It was possible to predict farrowing on the basis of increased activity in the validation dataset with a median of 8 h 51 min before the onset of farrowing. Alarms that indicated the need for confinement of the sow in a crate were generated with a median of 2 h 3 min before the onset of farrowing. These results suggest that the developed model should be sufficient to provide early warning of approaching farrowing and secondary alarm indicating the need to confine a sow in a crate.

## 1. Introduction

It is common practice in modern intensive pig husbandry to confine sows in farrowing crates, usually for four to five weeks, including at least a few days before the onset of farrowing. The main reason for this practice is to improve piglet survival rate by protecting newborn piglets from fatal or injurious crushing by the mother sow [1]. However, the confinement of sows in crates has a negative impact on the sows’ welfare, such as limited freedom of movement, limited social interactions with newborn piglets [2,3], and diminished health [4,5]. Confinement also prevents much of the prenatal nest-building behaviour, an essential part of the behavioural repertoire in sows, which starts approximately 24 h before parturition, is most intense 6 to 12 h before parturition, and then, decreases as parturition begins [6,7]. Increased physiological stress for the sow is a consequence of the confinement in a crate, which is indicated by changes in the hypothalamic–pituitary–adrenal (HPA) axis, consistent with chronic stress [8].

The concept of temporary crating was developed as a response to increased public concern about welfare of crated sows [9]. According to this concept, sows should be temporarily confined in farrowing crates only during the critical period of the piglets’ life, when piglet crushing is most probable, i.e., in the first days after farrowing [10,11]. When the crate is opened, the farrowing pen offers additional space for the sow, providing a compromise between the needs of the farmer, the sow, and her piglets [1]. This allows the sow to stay unconfined during the prenatal nest-building phase from 24 h ante partum until the approach of farrowing, which has a positive impact on the sow’s welfare [12]. It is challenging to choose the right moment to confine an individual sow in a farrowing crate under farm conditions in a way that makes nest building possible and does not increase the risk of piglet crushing. Due to the biological variability in gestation length, time-consuming observations of sows would be necessary. However, in many sows, confinement of sows in crates based on a calculated farrowing date of the batch could either disturb adequate nest-building behaviour or the farrowing process.

The activity level of a sow that builds a nest before farrowing increases due to hormonal changes and the presence of external stimuli [7,13,14]. This increase in activity level can be automatically detected with sensor technology. To date, three types of sensor technologies have been used for this purpose, i.e., infrared photocells, force sensors, and accelerometers. Infrared photocells and force sensors have been installed in farrowing crates and as the farrowing approached, the measured activity of sows increased slightly, then, more than doubled the day before farrowing [15,16]. Accelerometers were tested for measurement of activity of sows with sensors installed in collars and in tags [17,18,19]. Measurement of the activity of sows with accelerometers installed in collars indicated, similarly to other sensor technologies, an increase of activity 16 to 20 h before the onset of farrowing [19]. When an accelerometer was installed inside an ear tag, the increase in activity was detected 48 h before the onset of farrowing [17].

Automated detection of increases in sow activity with the use of sensor technology makes possible the prediction of the onset of farrowing. This could be useful in practical conditions to shorten surveillance intervals by farm staff, and the pen could be prepared for an optimal farrowing (e.g., providing nest-building material and activating a heating source) [17]. However, for selecting the optimal period of temporary crating of sows, there is a need for a reliable method to detect when an individual sow starts and finishes nest-building behaviour. We hypothesize that providing a “second-stage” alarm, indicating the end of nest-building behaviour, should ensure that farm staff confine a sow in a crate after nest building is finished but before farrowing starts. Then, the potential of farrowing pens with the possibility of temporary crating to achieve a compromise between the needs of the farmer, the sow, and her piglets could be realized.

Thus, the objective of this paper was to model the dynamics of acceleration data in a period before the onset of farrowing, in three types of farrowing pens, to automatically detect the beginning and end of nest-building behaviour. The technique developed should allow provision of a “first-stage” and “second-stage” alarm for the farm staff which should be especially relevant for improving sow welfare in pens with the possibility of temporary crating.

## 2. Materials and Methods

### 2.1. Ethical Statement

Project PIGwatch was authorized by the Ethical Committee of the Austrian Federal Ministry of Science, Research and Economy and by the Ethical Committee of Vetmeduni Vienna (GZ: BMWFV-68.205/0082-WF/II/3b/2014) according to the Austrian Tierversuchsgesetz 2012, BGBl. I Nr. 114/2012.

### 2.2. Experimental Setup

#### 2.2.1. Animals and Housing

The experiment was conducted between June 2014 and May 2016 at the pig research and teaching farm (VetFarm) of the University of Veterinary Medicine Vienna, Vienna, Austria. In total, 53 Austrian Large White sows and Landrace × Large White crossbreds sows were included in the experiment. The sows were kept in three types of farrowing pens with the possibility of keeping a sow either unconfined or in a farrowing crate. Out of 53 sows, 18 were kept in SWAP (Sow Welfare and Piglet Protection) pens (Jyden Bur A/S, Vemb, Denmark), 18 in trapezoid pens (Schauer Agrotronic GmbH, Prambachkirchen, Austria) and 17 in wing pens (Stewa Steinhuber GmbH, Sattledt, Austria). None of the animals included in the experiment was confined in a farrowing crate from the introduction to the farrowing pen until the end of farrowing.

**Figure 1:**
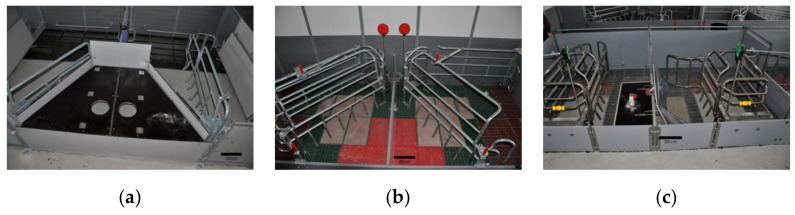
Farrowing pens with possibility of temporary crating. On the left side of each picture the crate is open. On the right side of each picture the crate is closed. (**a**) Adjacent pair of SWAP pens, (**b**) adjacent pair of trapezoid pens, and (**c**) Adjacent pair of wing pens.

The SWAP pens had an area of 6.0 m^2^. The pens had a solid concrete floor in the front (lying area) and a slatted cast iron floor in the back (defecation area). The pen had 2 troughs, one for the crated and one non-crated sow (Figure 1a). The trapezoid pens had an area of 5.5 m^2^. The pens had plastic flooring in the creep area and solid concrete flooring in the sow lying area in front of the trough (Figure 1b). The wing pens had an area of 5.5 m^2^. The pens were partly slatted with plastic elements and solid concrete elements (Figure 1c). In all 3 pen types a straw rack was mounted in the front area of the pen, in close proximity to the trough.

The sows were introduced to the farrowing pens approximately five days before the expected date of farrowing. The date was derived from the usual gestation length of sows (114 days), which could vary from 105 to 125 days [20]. As farrowings were not hormonally induced, it was uncertain when a sow would farrow. The experimental period was from the introduction of the sow to the farrowing room until the end of farrowing. The experimental pens were located in a testing unit of the VetFarm which had an automatic ventilation system. The average temperature in the room was 22 °C. The sows were fed twice a day during the experimental period. Water was provided permanently in the troughs via a nipple drinker or an automatic water-level system. To fulfil the need for adequate material to explore and for nest building, sows and piglets were offered straw in the aforementioned rack throughout their stay in the pens. The racks were half filled in the morning and whenever the racks were empty.

#### 2.2.2. Video Recording

The behaviour of sows was video recorded from introduction to the farrowing pens until weaning with two-dimensional (2D) cameras in order to create a dataset that could be labeled. Each pen was equipped with one IP camera (GV-BX 1300-KV, Geovision, Taipei, China) locked in protective housing (HEB32K1, Videotec, Schio, Italy) hanging 3 m above the pen, giving an overhead view. Additionally, infrared spotlights (IR-LED294S-90, Microlight, Bad Nauheim, Germany) were installed in order to allow night recording. The images were recorded with 1280 × 720 pixels resolution, in MPEG-4 format, at 30 fps.

The cameras were connected to a PC on which Multicam Surveillance System (8.5.6.0, Geovision, Taipei, China) was installed. The system allowed simultaneous recording of images from 9 cameras. The PC had a processor Intel5, CPU 3330, 3 GHz (Intel, Santa Clara, CA, USA) with 4 GB of physical memory. The operating system was Microsoft Windows 7 Professional (Redmond, WA, USA). Recordings were stored on exchangeable, external 2 and 3 TB hard drives.

#### 2.2.3. Data Labeling

Recorded videos were manually labeled in order to create a reference dataset on the basis of which further data analysis could be performed. In the first step of the labeling process, the time of the onset of farrowing of each individual sow (n = 53) was labeled. The onset of farrowing was defined as the point in time when the body of the first piglet born dropped on the floor. The time of birth of the last piglet indicated the end of farrowing. Labeling software Interact (version 9 and 14, Mangold International GmbH, Arnstorf, Germany) was used to label the images.

#### 2.2.4. Accelerometer System

The SMARTBOW^®^ system (Smartbow GmbH, Weibern, Austria) comprising of ear tags, wall points, and station delivered information on acceleration of sensors attached to the sows. The SMARTBOW^®^ ear tag was equipped with an accelerometer sensor that measures acceleration in 3 axes (xyz). The specification of the SMARTBOW^®^ system was described in detail in previous work by Oczak et al. [21]. There were five SMARTBOW^®^ antennas installed on the walls of the experimental compartment. Data loss was not more than 10% in the experimental period.

#### 2.2.5. Dataset

For the purpose of modeling of the acceleration data, 27 (50.9%) sows out of the total of 53 sows included in the experiment were chosen as a training set and 26 (49.1%) as a validation set (Table 1). The animals, both in the training set and in the validation set, were equally distributed between SWAP, trapezoid and wing pens. Comparison of statistical measures of effectiveness of the algorithm (e.g., median, 1st quartile, and 3rd quartile of time of alarms until onset of farrowing) on training and validation sets enabled conclusions to be drawn on the efficiency the algorithm to work on other independent datasets.

### 2.3. Model

#### 2.3.1. Input Variable

**Figure 2:**
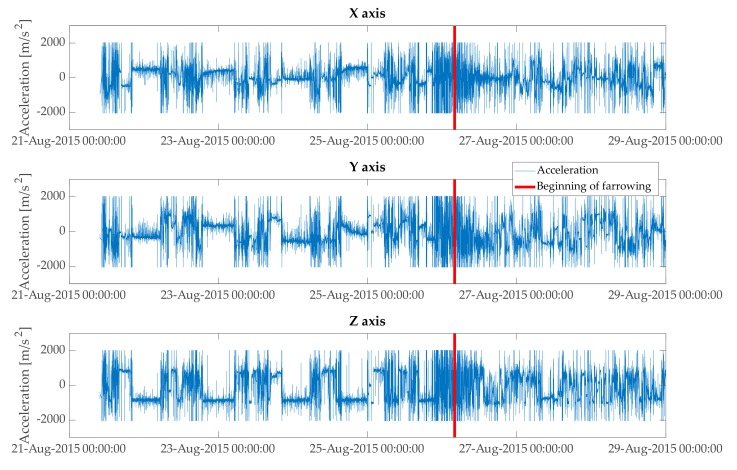
Raw axes of accelerometer data (**x, y, z**). Period depicted in the plot starts at the introduction of a sow to a farrowing pen and ends around 3 days after the beginning of farrowing.

**Figure 3:**
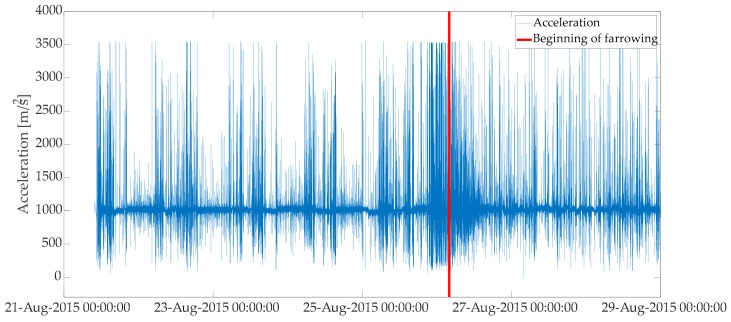
Total physical acceleration. Period depicted in the plot starts at the introduction of a sow to a farrowing pen and ends around 3 days after beginning of farrowing.

One variable was used as an input to estimate a model for prediction of farrowing and provision of the first- and second-stage alarms. In the initial step, total physical acceleration (magnitude) was estimated from three axes of the accelerometer data (xyz) (Figure 2 and Figure 3) with the equation:

(1) $$Acc\left(i\right)\;=\;\sqrt{x{\left(t\right)}^{2}+y{\left(t\right)}^{2}+\;z{\left(t\right)}^{2}},\;t=\;1,2,\;.\;.\;.\;,\;N$$

In the above equation $Acc\left(t\right)$ is total physical acceleration at a given time point t. Three axes of the accelerometer data are represented with $x\left(t\right),\;y\left(t\right)$, and $z\left(t\right)$ at a given time point t.

**Figure 4:**
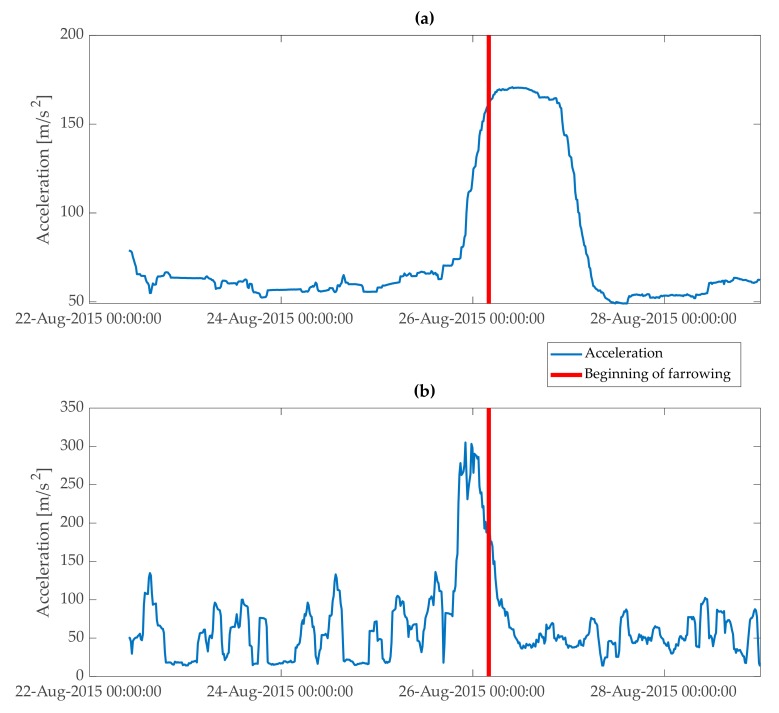
Standard deviation calculated on acceleration data with two sizes of sliding windows. Period depicted in the plot starts at the introduction of a sow to a farrowing pen and ends around 3 days after beginning of farrowing. (**a**) Sliding window of 24 h with 15 min steps. Variation in activity related to diurnal rhythms was filtered out. Two rapid changes in trend in activity were at the beginning of nest-building behaviour and at the end of nest-building behaviour, when this sow started farrowing. (**b**) Sliding window of 2 h with 15 min steps. Peak of activity was a few hours before the onset of farrowing.

Total physical acceleration (magnitude) was smoothed with standard deviation calculated on a sliding window of 24 h with 15 min steps. A smoothing window of 24 h eliminated the variation in activity of animals related to diurnal rhythm. Steps of 15 min allowed the sampling to be frequent enough for the proposed application (Figure 4). The first 12 h of smoothed total physical acceleration were not used as an input to the model, because the activity of a sow in the first hours after introduction to a farrowing pen was increased due to exploration of the new environment (Figure 4). These first hours were eliminated to avoid variation in the input variable not related to the approaching farrowing or nest-building behaviour of sows.

#### 2.3.2. Kalman Filtering and Fixed Interval Smoothing (KALMSMO) Algorithm

Input variable (Figure 4a) was used to estimate a trend in activity of each sow. Changes in trends (dynamics) in activity of sows were the basis for detection of approaching farrowing and provision of first- and second-stage alarms. To estimate the dynamics of activity of sows the Kalman filtering and fixed interval smoothing (KALMSMO) algorithm was used [22]:

Prediction:

(2) $$\hat{x}\left(k|k-1\right)\;=\;A\hat{x}\left(k-1\right)$$

(3) $$\;P\left(k|k-1\right)\;=\;AP\left(k-1\right)A^{T}\;+\;DQ_{nvr}D^{T}\;$$

Correction:

(4) $$\hat{x}\left(k\right)\;=\;\hat{x}\left(k|k-1\right)+g\left(k\right)\{y\left(k\right)-h^{T}\left(k\right)\hat{x}(k|k-1)\}$$

(5) $$\;\;g\left(k\right)\;=\;P\left(k|k-1\right)h\left(k\right){\left[1+h^{T}\left(k\right)P\left(k|k-1\right)h\left(k\right)\right]}^{-1}\;\;$$

(6) $$P\left(k\right)\;=\;P\left(k|k-1\right)-g\left(k\right)h^{T}\left(k\right)P(k|k-1)$$

(7) $$\;\;P^{*}\left(k\right)\;=\;\sigma^{2}\left(k\right)P\left(k\right)\;\;$$

Smoothing:

(8) $$\hat{x}\left(k|N\right)\;=\;\hat{x}\left(k\right)-P^{*}\left(k\right)A^{T}\lambda \left(k\right)$$

with

(9) $$\;\;\lambda \left(N\right)\;=\;0\;\;$$

(10) $$\;\;P^{*}\left(k|N\right)\;=\;P^{*}\left(k\right)+P^{*}\left(k\right)AP^{*}{\left(k+1|k\right)}^{-1}\;\;\;\left[P^{*}\left(k+1|N\right)-P^{*}\left(k+1|k\right)\right]P^{*}{\left(k+1|k\right)}^{-1}AP^{*}\left(k\right)$$

In the above equations $\hat{x}\left(k|k-1\right)$ is the predicted value of the input variable (acceleration) at a given time point k considering the value of the input variable at previous time point k−1. A and D are matrices:

$$A\;=\;\left[\begin{array}{cc} \alpha & \beta \\ 0 & \gamma \end{array}\right],\;D\;=\;\left[\begin{array}{cc} \delta & 0\\ 0 & \varepsilon \end{array}\right]\;$$

where matrices comprise as special case the Integrated Random Walk (IRW: α = β = γ = ε = 1, δ = 0) and $Q_{nvr}$ is a noise to variance matrix that can be specified by the user of the algorithm. $P\left(k|k-1\right)$ is a predicted value of error covariance matrix P at a given time point k considering values of error covariance matrix at previous time point k−1  ; $y\left(k\right)$ is the actual value of the input variable (acceleration) at a given time point k; $h\left(k\right)$ is a vector that relates the scalar observation y(k) to the parametric state variables at a given time point k; $\sigma^{2}\left(k\right)$ is error variance at a given time point k obtained in a three-stage procedure described in Young [22]; $\lambda \left(k\right)$ is a vector, a Lagrange multiplier term required in the solution of the optimization problem in order to ensure that the estimate is obtained under the constraint that the parameter vector evolves according to the state equations of Kalman Filter; $P^{*}\left(k\right)$ is a matrix with standard errors of the estimated value of the input variable (acceleration) at a given time point k.

#### 2.3.3. Data Analysis

**Figure 5:**
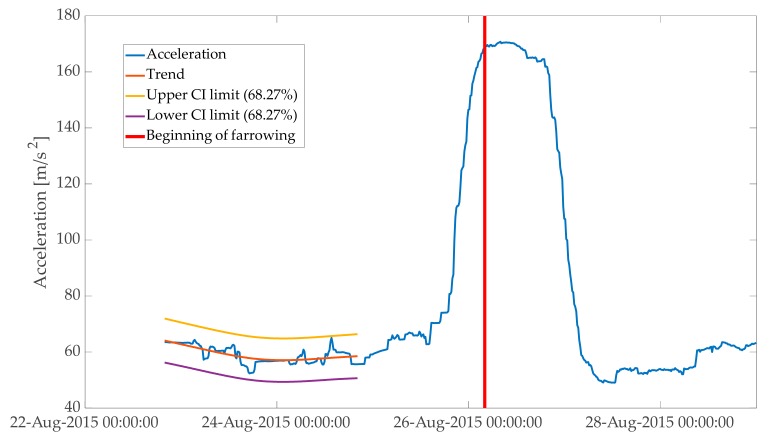
Activity trend estimated in the first 48 h after the first 12 h of the input variable was removed. Fixed interval on input variable for KALMSMO algorithm ends 31 h before the onset of farrowing. Period depicted in the plot starts 12 h after introduction of a sow to a farrowing pen and ends approximately 3 days after beginning of farrowing.

**Figure 6:**
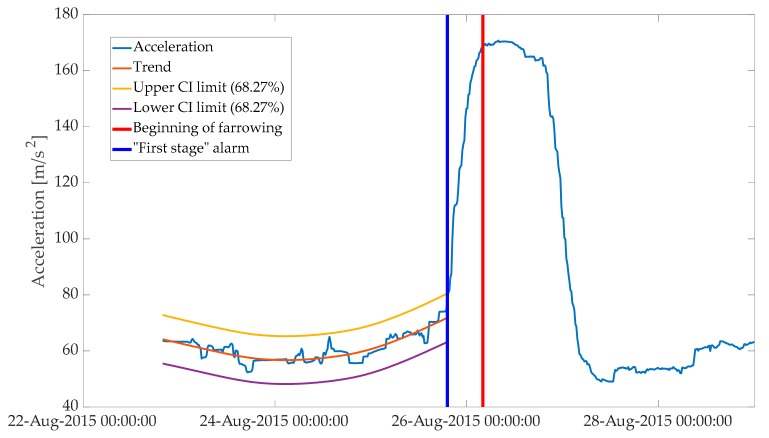
The “first-stage” alarm, at 9 h, before the onset of farrowing. Period depicted in the plot starts 12 h after introduction of a sow to a farrowing pen and ends around 3 days after farrowing.

**Figure 7:**
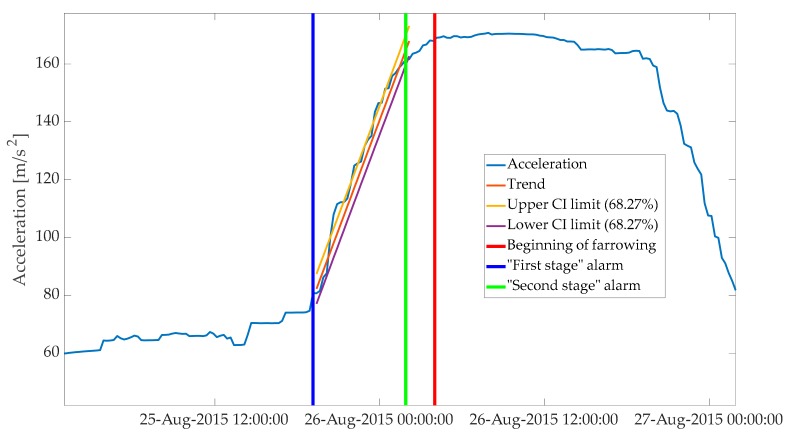
The “second-stage” alarm, at 2 h, before the onset of farrowing. Period depicted in the plot starts around 24 h before the onset of farrowing and ends around 20 h after it.

The KALMSMO algorithm was fitted to an input variable at a fixed interval of 48 h to estimate a level of activity for each sow in a period when the animal was not preparing for farrowing (Figure 5). Noise to variance ratio ($Q_{nvr})$ was set to 0.000001 to adjust the estimated trend to dynamics of the sow’s activity. Figure 5, Figure 6 and Figure 7 in Materials and Methods explain the methodology used in this research.

Next, the fixed interval was expanded recursively by 15 min steps until the trend in animal activity changed to significantly increasing. This was indicated by the input variable reaching a higher value than the upper confidence interval of the estimated trend (Figure 6). At this time point, the “first-stage” alarm was generated, which indicated that farrowing was approaching.

For the next step, the KALMSMO algorithm was fitted to the acceleration data on a fixed interval, starting from the time point of the “first-stage” alarm. In the consecutive steps, the fixed interval was expanded recursively by 15 min steps until the trend in animal activity changed to significantly decreasing. This was indicated by the input variable reaching a lower value than the lower confidence interval of the estimated trend (Figure 7) and, at this time point, the “second-stage” alarm was generated. This alarm could be interpreted as an indication that nest-building behaviour had ending.

Contrary to Manteuffel et al. [23] and Pastell et al. [14] we decided not to use accuracy, sensitivity, and specificity as measures of performance of the algorithm that was developed in this research. Instead, we decided to mainly evaluate the performance of the algorithm on the basis of the duration between the time when an alarm was generated and the onset of farrowing, similar to Traulsen et al. [17] and on the basis of the associated distribution of alarms. In our opinion, accuracy, sensitivity, and specificity as measures of algorithm performance are difficult to interpret when it comes to farrowing prediction, especially when different authors use various definitions of “true positive” alarms [13,14,23] and, accordingly, there is no consensus at what time before farrowing “true positive” alarm occurs.

Confidence interval bounds were tested on the training set for the first and second stage of fitting of KALMSMO algorithm (38.29%, 68.27%, 86.64%, 95.45%, 98.76%, 99.73%, 99.95%, 99.99%, 99.999%, and 99.9999%). The first stage of model fitting resulted in the “first-stage” alarm, while the second stage of model fitting resulted in the “second-stage” alarm. With narrower bounds of confidence intervals, the alarms in the first and second stage of fitting could have occurred too early or were false, while wider bounds could have resulted in too late or no alarms. Optimal confidence interval bounds, for the “first stage” of model fitting, were set, based on the median and interquartile range of duration between the time when the “first-stage” alarm was generated and the onset of farrowing. Additionally, the following characteristic values were considered: the number of “first-stage” alarms that occurred earlier than 48 h before onset of farrowing, the number of “first-stage” alarms generated after the onset of farrowing, and the number of sows for which no “first stage” alarms were generated.

The preferred time frame for the “first-stage” alarms was within 48 h before the onset of farrowing, and the alarm was not supposed to be generated after the onset of farrowing. This was decided, on the one hand, because according to our experience and scientific literature, sows do not start preparing for farrowing (nest-building behaviour) earlier than 48 h before the onset of farrowing [7]. On the other hand, “first-stage” alarms which were generated after the onset of farrowing would not be useful for the user of a farrowing prediction system.

Similar to “first stage” model fitting, in the “second stage”, values of optimal confidence interval bounds were set based on median and interquartile range of duration between the time when the “second-stage” alarm was generated and the onset of farrowing. Additionally, the following characteristic values were considered: the number of “second-stage” alarms that occurred earlier than 48 h before onset of farrowing and the number of sows for which no “second-stage” alarm was generated. To decide on the values of optimal confidence interval bounds in the “second stage” of model fitting, the number of “second-stage” alarms generated after the onset of farrowing was not significant. The reason for this was that we expected that many “second-stage” alarms would have been generated very close to the onset of farrowing, including many occurring after the onset of farrowing.

The preferred time frame for the “second-stage” alarm was after the “first stage” alarm (within 48 h before the onset of farrowing) and not later than the end of farrowing. We decided on this range of time because we expected that the “second-stage” alarm was related to the time when the sow had already finished the farrowing preparation (end of nest-building behaviour) and the trend in its activity had already decreased. The methodology for modeling data to raise the “second-stage” alarm prevented the alarms from being generated before the “first-stage” alarms. Although, when the “first-stage” alarm was generated, more than 48 h before the onset of farrowing, the “second-stage” alarm could have been generated after the “first-stage” alarm but still earlier than 48 h before the onset of farrowing. From the point of view of an on-farm user, the optimal time for the “second-stage” alarm would be just before the onset of farrowing (1 to 2 h). However, in many sows, because a high activity level could be observed not only many hours before the onset of farrowing but also when first piglets were already born [24], we decided that the preferred time frame of the “second-stage” alarm was after the onset of farrowing but before the end of farrowing.

Analysis was performed with a commercial software package (MATLAB 2019b, The MathWorks, Inc., Natick, MA, USA) and function irwsm of CAPTAIN toolbox [25] was used to fit the KALMSMO algorithm.

## 3. Results

**Figure 8:**
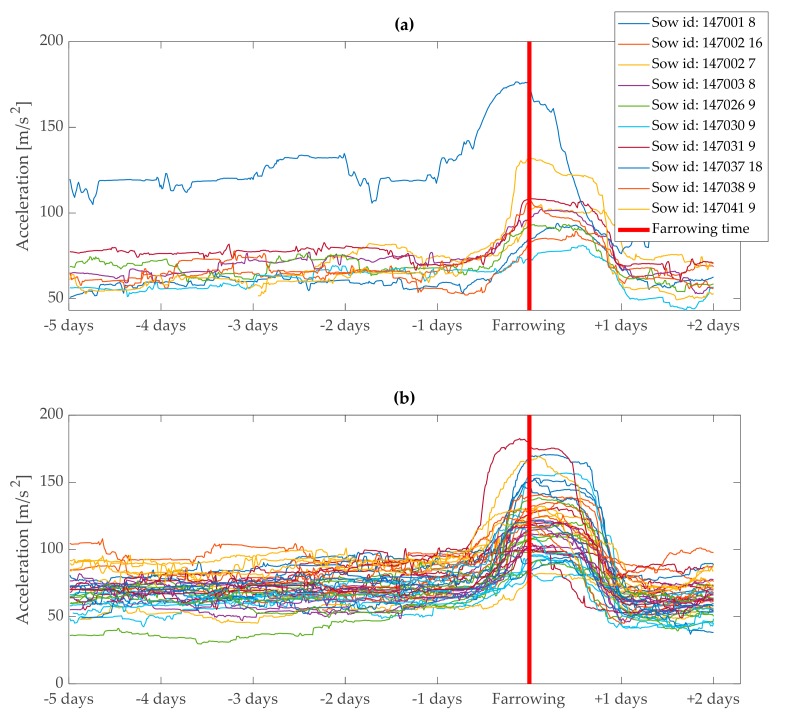
Activity of sows in a period from 5 days before to 2 days after onset of farrowing expressed as total physical acceleration (magnitude) smoothed with standard deviation, sliding window of 24 h with 15 min steps. Initial baseline activity level varied from around 40 to 120 (m/s^2^). Dynamics of feature variable before the onset of farrowing was similar between sows. (**a**) Activity of 10 sows out of 53 is presented for better visibility and (**b**) the activity of remaining 43 sows out of 53.

**Figure 9:**
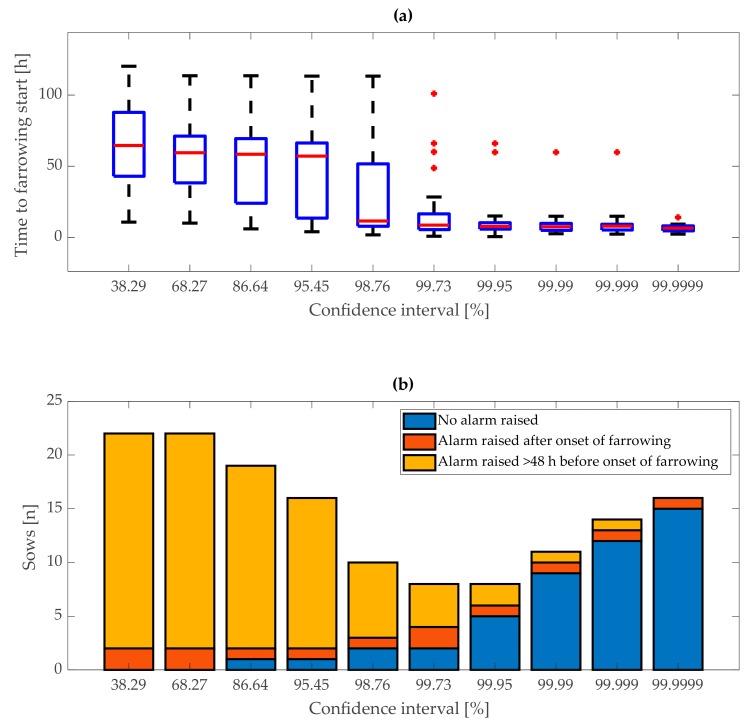
“First-stage” alarms in relation to confidence interval bounds on training dataset: (**a**) Distribution of duration between time of alarms and onset of farrowing and (**b**) the number of sows with no alarm generated, alarms generated earlier than 48 h before the onset of farrowing, and alarms generated after the onset of farrowing.

The KALMSMO algorithm was fitted recursively to the acceleration data of 27 sows in the training dataset to generate alarms indicating approaching farrowing (Figure 8). The fitting procedure was repeated with increasing confidence interval bounds from 38.29% to 99.9999%. As confidence interval bounds increased from 38.29% to 99.9999%, the median duration between the time when the “first stage” alarm was generated and the onset of farrowing, decreased from 64 h 31 min to 6 h 26 min (Figure 9). This was due to the fact that lower confidence interval bounds mean narrower threshold bounds for the model.

Changes in activity not related to approaching farrowing can raise the number of false alarms when a narrow threshold is crossed. However, health problems that could cause lower activity of animals, such as lameness, should not result in earlier alarms as “first-stage” alarms were generated only when the upper confidence interval bound was crossed. Nevertheless, with lower confidence interval bounds, the “first-stage” alarms were generated after sows entered farrowing pens and further away from the onset of farrowing. The biggest change in median duration, between the time when the “first-stage” alarm was generated and the onset of farrowing, was between confidence interval bounds of 98.45% and 98.76%. The median decreased from 57 h 4 min to 11 h 29 min (Figure 9a).

The higher median of the duration between the time when a “first-stage” alarm was generated and the onset of farrowing, indicated that approaching farrowing was detected earlier. Earlier detection of approaching farrowing means that the model is more sensitive to behavioural changes of the sow. Additionally, earlier alarms give more time for the farmer to intervene before farrowing starts (e.g., preparation of the farrowing pen for farrowing). A very high median (e.g., >24 h) could be caused by false alarms, due to detection of behavioural changes that were not related to approaching farrowing.

The values of the interquartile range of the duration between the time when an alarm was genereated and the onset of farrowing formed two groups depending on the value of confidence interval bounds as follows: one with higher values of 52 h 47 min to 32 h 51 min for confidence interval bounds between 38.29% and 98.76%, and the second with lower values of 11 h 8 min to 3 h 26 min for confidence interval bounds between 99.73% and 99.9999% (Figure 9a).

A lower interquartile range was an indicator that “first-stage” alarms were generated at similar intervals before the onset of farrowing. This suggests that alarms were generated because of changes in sow behaviour related to one cause, specifically approaching farrowing and not because of other reasons. Thus, when deciding on optimal confidence interval bounds, the group with lower interquartile range was preferred.

In addition to the median and interquartile range of the duration between the time when an alarm was generated and the onset of farrowing, the following characteristic values were considered: the number of “first-stage” alarms that occurred earlier than 48 h before the onset of farrowing, the number of “first-stage” alarms generated after the onset of farrowing, and the number of sows for which no “first-stage” alarms were generated. The number of “first-stage” alarms that were generated earlier than 48 h before the onset of farrowing decreased from 20 (74.07%) to zero sows, as confidence interval bounds increased from 38.29% to 99.9999%. The number of sows with “first-stage” alarms generated after the onset of farrowing stayed between two (7.41%) and one (3.7%), and the number of sows for which no “first stage” alarms were generated increased from zero to 15 (55.56%). A decreasing trend in the number of “first-stage” alarms generated earlier than 48 h before the onset of farrowing meant that as confidence interval bounds increased, alarms were generated nearer to the onset of farrowing. Simultaneously, for more and more sows no alarm was generated (Figure 9b).

The sum of “first-stage” alarms that occurred earlier than 48 h before onset of farrowing, “first stage” alarms generated after the onset of farrowing, and sows for which no “first stage” alarms were generated was the lowest (n = 8, 29.63%) for the confidence interval bounds of 99.73% and 99.95%. For both of these confidence interval bounds, the median of duration between the time when the “first-stage” alarms were generated and the onset of farrowing were similar (8 h 36 min and 7 h 35 min). The interquartile range was more than double for a confidence interval bound of 99.73% than for 99.95% (11 h 8 min and 4 h 28 min). This suggests that, for a confidence interval bound of 99.95%, alarms were generated more consistently around the time of farrowing than for the confidence interval bound of 99.73%. Thus, we decided that the confidence interval bound of 99.95% is optimal for the first stage of model fitting (Figure 9b).

**Figure 10:**
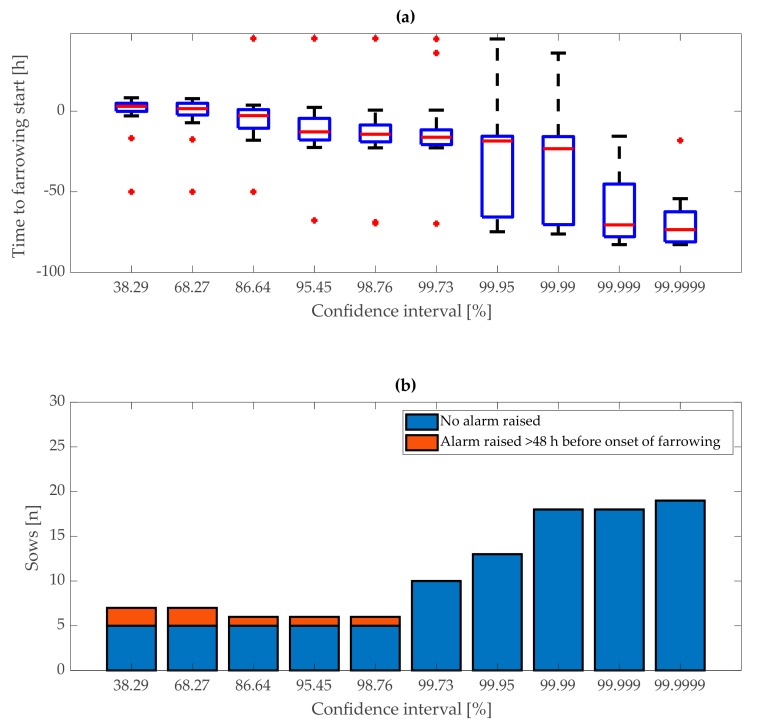
“Second-stage” alarm in relation to confidence interval bounds on training dataset. (**a**) Distribution of duration between time of alarms and onset of farrowing and (**b**) the number of sows with no alarm generated and alarms generated earlier than 48 h before the onset of farrowing.

In the second stage, the KALMSMO algorithm was fitted to the acceleration data from the time point of the “first-stage” alarms, where “first-stage” alarms were generated on the optimal 99.95% confidence interval bound. The KALMSMO algorithm, in the second stage, was also fitted with increasing confidence interval bounds from 38.29% to 99.9999%. As confidence interval bounds increased, the median duration between the time when a “second-stage” alarm was generated and the onset of farrowing, decreased from 2 h 58 min before the onset of farrowing, to 73 h 33 min after the onset of farrowing. Thus, similar to “first stage” model fitting, narrower confidence interval bounds resulted in earlier “second-stage” alarms. The interquartile range of “second-stage” alarms increased from 5 h 1 min to 54 h 32 min (Figure 10a).

The number of alarms generated earlier than 48 h, before the onset of farrowing, decreased from 2 (7.41%) to zero, as the confidence interval changed. The number of sows for which no alarm was generated was five (18.52%) for confidence intervals between 38.29% and 98.76%. For confidence intervals above 98.76%, the number of sows for which no alarm was generated, increased to 19 (70.37%).

The aim of algorithm fitting, in the “second stage”, was to provide alarms mainly before the onset of farrowing. Thus, optimal confidence interval bounds were selected, and therefore the median duration of “second-stage” alarms was before the onset of farrowing. This condition was met only for confidence interval bounds of 38.29% and 68.27%. The sum of no alarms and the alarms generated earlier than 48 h before the onset of farrowing was seven (25.93%) for both of these confidence interval bounds, which was low as compared with the result of the other confidence interval bounds. Only for confidence interval bounds of 86.64% to 98.76%, this value was lower at six (22.22%). The interquartile range was lower for a confidence interval of 38.29% (5 h 1 min) than 68.27% (7 h 16 min). A lower interquartile range was an indication that “second-stage” alarms were generated at similar intervals before the onset of farrowing. This suggested that “second-stage” alarms based on confidence interval bounds of 38.29% were generated more likely because of changes in sows’ behaviour related to approaching farrowing rather than because of other reasons. Thus, the confidence interval bounds of 38.29% were selected as optimal for “second stage” model fitting.

**Figure 11:**
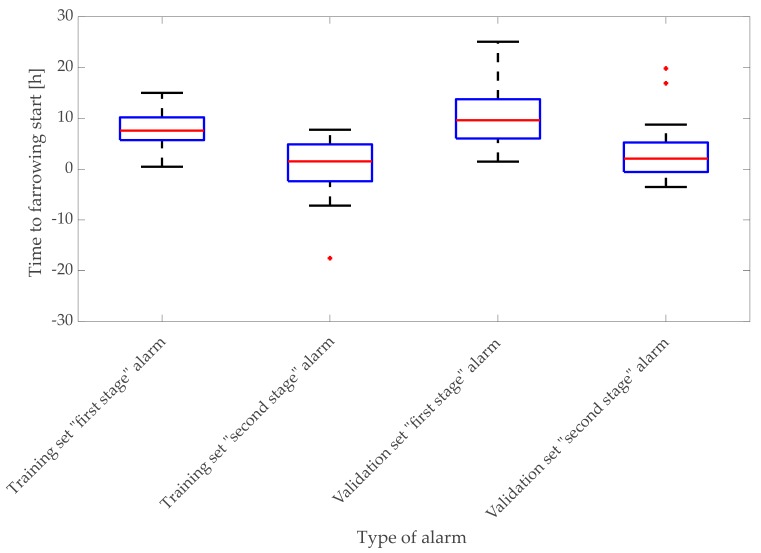
Distribution of duration between time of alarms and onset of farrowing in training and validation datasets.

Optimal confidence interval bounds selected on the basis of the training dataset were validated on a group of 26 sows. The distribution of first- and second-stage alarms was similar in both datasets. In the training set, the median of “first-stage” alarms was 7 h 35 min before the onset of farrowing with 1st quartile of 5 h 44 min and 3rd quartile of 10 h 12 min, whereas, in the validation set, the median of “first-stage” alarms was 9 h 38 min with 1st quartile of 6 h 3 min and 3rd quartile of 13 h 46 min (Figure 11).

In the training set, the median of “second-stage” alarms was 1 h 32 min before the onset of farrowing with 1st quartile of 2 h 23 min after the onset of farrowing and 3rd quartile of 4 h 53 min before the onset of farrowing, whereas in the validation set the median of “second-stage” alarms was 2 h 4 min before the onset of farrowing with 1st quartile of 34 min after the onset of farrowing and 3rd quartile of 5 h 15 min before the onset of farrowing (Figure 11).

The distribution of first- and second-stage alarms in both training and validation datasets indicates that the “second-stage” alarms occurred later than the “first stage” alarms (Figure 11). However, for some of the animals in the training and validation datasets, alarms were not generated or were not generated in the preferred period. Specifically, in the training dataset, “first-stage” alarms were not generated, and “second-stage” alarms were generated for five (18.52%) animals (Table 2). If no “first-stage” alarm was generated for a certain animal, also no “second-stage” alarm was generated for this animal, both in training and in validation datasets. In the validation set, no first- and second-stage alarms were generated for eight (30.77%) animals (Table 3). The alarms that were generated outside of the desired period in the “first stage” were alarms generated after the onset of farrowing and earlier than 48 h before the onset of farrowing. The alarms that were generated outside of the desired period in the “second stage” were alarms generated earlier than 48 h before the onset of farrowing and alarms generated after the end of farrowing. In the training dataset, there was one (3.7%) “first-stage” alarm generated after the onset of farrowing, two (7.41%) alarms generated earlier than 48 h before the onset of farrowing, two (7.41%) “second-stage” alarms generated earlier than 48 h before the onset of farrowing, and four (14.81%) “second stage” alarms generated after the end of farrowing (Table 2). In the validation dataset, there were four (14.81%) “second-stage” alarms generated after the end of farrowing (Table 2).

Out of 27 sows in the training dataset, for 19 (70.37%) sows, a “first-stage” alarm was generated in a period of 48 h before the onset of farrowing until the onset of farrowing. For 16 (59.96%) animals, a “second-stage” alarm was generated 48 h before the onset of farrowing until the end of farrowing. Out of 26 sows in the validation dataset, for 18 (69.23%), a “first-stage” alarm was generated in a period of 48 h before the onset of farrowing until the onset of farrowing. For 17 (65.38%) animals, a “second-stage” alarm was generated over a period of 48 h before the onset of farrowing until the end of farrowing (Table 3).

The median of “first-stage” alarms in SWAP pens was 7 h 45 min before the onset of farrowing with a 1st quartile of 5 h 35 min and a 3rd quartile of 11 h 59 min. In the trapezoid pens, the median of “first-stage” alarms was 7 h 48 min before the onset of farrowing with a 1st quartile of 6 h 3 min and a 3rd quartile of 10 h 12 min. Finally, in wing pens the median of “first-stage” alarms was 10 h 36 min before the onset of farrowing with a 1st quartile of 5 h 44 min and a 3rd quartile of 15 h 1 min.

The median of “second-stage” alarms in SWAP pens was 1 h 09 min before the onset of farrowing with a 1st quartile of 3 h 21 min after the onset of farrowing and a 3rd quartile of 3 h 2 min before the onset of farrowing. In the trapezoid pens, the median of “second stage” alarms was 3 h 9 min before the onset of farrowing with a 1st quartile of 34 min after the onset of farrowing and a 3rd quartile of 5 h 5 min before the onset of farrowing. Finally, in the wing pens, the median of “second-stage” alarms was 1 h 46 min before the onset of farrowing with a 1st quartile of 22 min after the onset of farrowing and a 3rd quartile of 6 h 48 min after the onset of farrowing.

**Figure 12:**
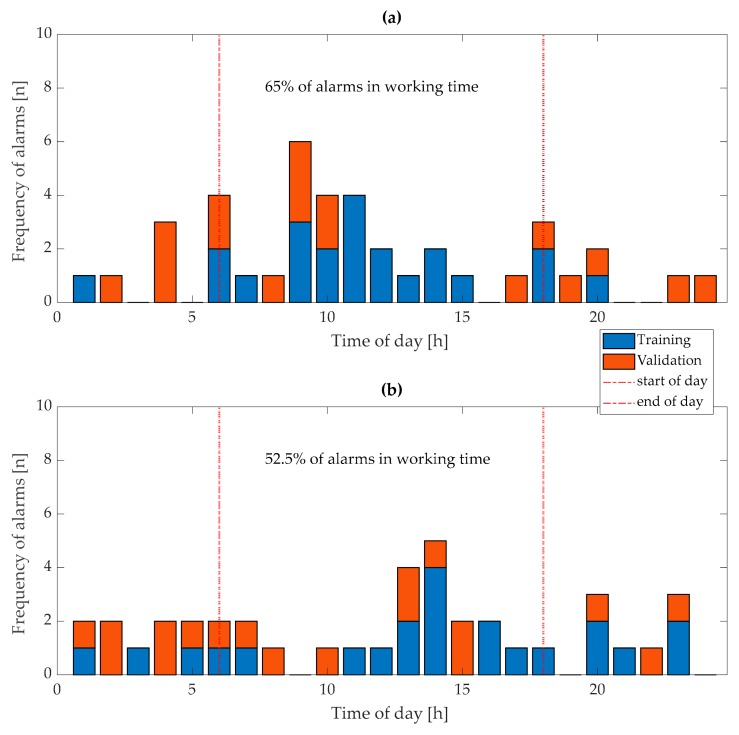
Distribution of time of alarms according to time of day: (**a**) “First-stage” alarms and (**b**) “second-stage” alarms.

Assuming that the standard working time of farm staff in farrowing compartments starts at 6:00 and ends at 18:00, 65% (26) of “first-stage” alarms and 52.5% (21) of “second-stage” alarms in training and validation sets were generated when farm staff were present to assist with the farrowing process (Figure 12).

## 4. Discussion

In this research we considered pigs as CITD systems, which stands for complex, individually different, time-varying, and dynamic (CITD) systems [26]. The individually different character of pigs, as any other living organisms, can be observed in their differing baseline of activity levels. All animals are individually different in their responses [27]. In some animals, the baseline activity measured by ear mounted accelerometers was double that of others (Figure 8). These differences in activity level between individuals are also related to time varying characteristics of living organisms. An animal response to a stimulus or stressor could be different each time it happens. An animal is constantly looking for a good energy balance and, consequently, is continuously changing its physical and mental status [27]. In the study by Manteuffel et al. [23], an improvement of the quantitative prediction of farrowing was achieved by using a different approximation function for very young sows. Thus, the dynamics of activity can change in the same animal over time as it grows older. In addition to individual differences between animals, factors such as breed [28] and environment [29] can affect the activity of sows on a group level, which makes modeling animal activity for the purpose of farrowing prediction more challenging. However, the objective of this study was to model the data for real-time monitoring, despite differences in activity between individual animals.

For this purpose, a KALMSMO model was individually and recursively fitted to acceleration data. Then, changes in dynamics of the activity of sows were detected as an increased and decreased trend in acceleration data. Such an individual modeling approach, in which models adapt to the activity of individuals, was also applied for prediction of farrowing in research by Pastell et al. [14], Traulsen et al. [17], and Manteuffel et al. [23], whereas in research by Oczak et al. [13], Oliviero et al. [16], and Cornou and Kristensen [30] group level modeling was applied. Individual modeling of sows’ activity for farrowing prediction has an advantage, because the performance of such models depends on similarity in dynamics of sows’ activity and is independent from baseline activity level of the animals. Thus, performance of these models should be similar when validated on independent datasets such as multiple pig sites with various breeds and housing conditions.

In previous research on farrowing prediction, the main objective was to predict the onset of farrowing as accurately as possible [14,17,23]. The basis for estimation of onset time of farrowing was the increase of sows’ activity related to nest-building behaviour [7,15]. In our research, increase in the activity level of sows was modeled to provide “first-stage” alarms. The median duration, before the onset of farrowing, of “first-stage” alarms in our validation set, was 9 h 38 min with a 1st quartile of 6 h 3 min and a 3rd quartile of 13 h 46 min. This result was similar to the results of a study by Pastell et al. [14] in which accelerometers were attached to neck collars. In the study by Pastell et al. [14] the median duration of alarms before the onset of farrowing was around 13 h with a slight variation of 30 min depending on adjustment of model parameters. Traulsen et al. [17] reported that for 84.2% of all sows equipped with a SMARTBOW^®^ accelerometer sensor, farrowing was successfully predicted; only one of the animals was outside the 12 h window before the onset of farrowing. This does not accurately compare with our results but suggests that the outcome was similar to ours and to the result of Pastell et al. [14]. In research by Manteuffel et al. [23], who used light barriers for farrowing prediction, the best performing qualitative prediction generated alarms with a 1st quartile of 13 h and a 3rd quartile of 20 h before the onset of farrowing. This result seems to indicate that alarms were generated earlier than in our study. However, the authors also reported a shorter duration between alarm and onset of farrowing for alternative models, which had a poorer performance when evaluated with accuracy, sensitivity, and specificity. The reason for the difference in results between our study and Manteuffel et al. [23] could have been the different methodology of evaluation of algorithm performance. Additionally, different sensors were used, and sows were kept confined in crates during the experiment, while in our experiment sows were allowed to move freely in the pen. In our own previous study, in which the SMARTBOW^®^ accelerometer senor was used, alarms were generated, on average, 11 h before the onset of farrowing [13].

The practical application of “first-stage” alarms, generated approximately 6 to 13 h before the onset of farrowing, could be to warn the farmer about approaching farrowing in an automated way. This could reduce labor costs otherwise required for regular control of sows in farrowing compartments. It should also limit the need to get in close contact with sows which could interrupt the sow’s activities at a time when she is sensitive to outside disturbances [16].

Austrian legislation requires that in the week before farrowing, the animals should be provided with suitable nest-building material but only when the slurry system allows it [31]. Thus, when the “first stage” alarm is generated “nest-building” material could be provided to the sow. Providing nest-building material in higher amounts after the “first-stage” alarm is generated or only after this alarm could limit the risk of blocking the slurry system with nest-building material and improve welfare of sows. Additionally, the procedure for preparing a farrowing pen for newborn piglets could be started when a “first-stage” alarm was generated [17]. However, when considering practical uses of such a monitoring system, the time of day when alarms are geknerated also has to be taken into account. In our study, only 65% (26) of the “first-stage” alarms were generated between 6:00 and 18:00. This raises a question of how alarms that were generated when no staff were present at the farm can be used.

According to the Austrian Animal Welfare Directive, which becomes mandatory in 2033, sows should only stay confined in farrowing crates during the “critical period” of the piglets’ life [11]. In order to reduce piglet crushing, farmers should be permitted to confine sows in farrowing crates from the onset of farrowing to a few days after farrowing. To provide sows with space for nest-building without increasing the risk of piglets getting crushed, crating should start after nest-building is finished and before farrowing starts. The crate has to be opened four days after farrowing [32]. “First-stage“ alarms, generated on the basis of increased activity of sows, provide information when nest-building behaviour starts. The time of the beginning of nest-building behaviour could be used to approximate when a sow will start to farrow. In our study, 61% (11 out of 18) of “first-stage” alarms in the validation dataset were generated between 6 and 13 h before the onset of farrowing. This variability in duration of nest-building behaviour was also apparent in our previous study in which different nest-building behaviours were accurately labeled [13]. Out of eight animals included in our previous study, seven had a clear peak of nest-building behaviour and in one animal it was not possible to distinguish the peak. Factors such as age or weight could influence motivation for nest-building behaviour [23]. Some sows had the peak of nest-building behaviour already 8 h before the onset of farrowing, while other sows peaked only 2 h pre partum. Thus, confining sows in crates just on the basis of the time of beginning of nest-building behaviour could leave many sows at risk of staying in crates during the nest-building phase and in many cases also during the peak of nest-building behaviour, which should be avoided. Quantitative prediction of onset of farrowing allows prediction of farrowing with a prediction error of approximately 4.5 h and with a standard deviation ranging from 5 h to 7.5 h [23]. Confining sows in crates on the basis of this prediction would also leave many sows at risk of staying in crates during the nest-building phase.

In this study, the method used to generate “second-stage” alarms is based on the change in trend in sow activity when nest-building behaviour ends. This change in trend is visible as “flattening” of the feature variable extracted from the acceleration data (Figure 4 and Figure 8). An advantage of this method is that when the “second-stage” alarm is generated, most of the nest-building activity of a sow should be finished. Thus, confining a sow, after the “second-stage” alarm was generated, should create little risk for sows staying in crates during nest-building, and especially during the peak of nest-building behaviour.

In order to select the right time to confine a sow in a crate, it also has to be considered that many sows still perform nest-building behaviour after the onset of farrowing, when the first piglet is already born [24,33]. It seems that this finding was confirmed in our research, because out of 18 “second-stage” alarms generated in the validation dataset, five (28%) were generated after the onset of farrowing. Thus, the “critical period” of piglets’ lives, as mentioned in the Austrian Animal Welfare Directive [11], could overlap with the time of nest-building behaviour at least for some sows.

In research on farrowing prediction [14,17,23], including this and our previous study [13] the onset of farrowing was defined as the birth of first piglet. This event is easily observable in a farrowing pen and was also commonly used for defining the onset of farrowing in scientific literature [34]. However, uterine contractions increase in frequency on average already 6 h before the first piglet is born together with an elevation of oxytocin levels [7]. Thus, it is expected that most “second-stage” alarms, with a median of 2 h 4 min before the onset of farrowing, would be generated when a sow experiences uterine contractions before the first piglet is born. Confining a sow in a crate in most situations involves moving the animal from one location in a farrowing pen to another, which is stressful for the animal if the farrowing process has already started. The influence of this human intervention on sow welfare has to be also taken into account when considering the right time to confine a sow in a crate.

Similar to “first-stage” alarms, many (47.5%) “second-stage” alarms were generated between 18:00 and 6:00 when mostly there would be no staff present on farms. Assuming that a practical application of developed model would be to confine sows in crates just after the “second-stage” alarm is generated, many of the sows that farrow in the night would stay unrestrained during farrowing.

This leads to the question of whether the application of a monitoring tool, which can be used effectively only in half of farrowing animals, is justified under practical conditions. We would hypothesise that most farmers would keep the sows confined in crates over night after they have received a “first-stage” alarm, in many cases in time for nest-building behaviour, as when the “second-stage” alarm is generated during the night they would not be able to intervene in the farrowing compartment. This suggests that the next step in our research should be to implement the developed model in a real-time system and verify how it could be used under practical conditions. Additionally, automated confinement of sows, without the need of human intervention is an interesting topic for further research.

Considering that for eight out of 26 sows (30.8%) no “first-stage” alarms were generated in our study, in future research we plan to include additional information on the health status of animals. Health problems such as lameness could affect the activity of sows and this could result in less “first-stage” alarms. In order to improve the performance of farrowing prediction we also plan to automatically detect and differentiate between behaviours that constitute nest-building behaviour i.e., rooting, pawing, and manipulation of pen or crate [13]. Such capability could provide more detailed information on sows’ behaviour and possibly also increase the performance of developed models. This is more likely to be successful with image analysis techniques, which have been applied for detection of complex behaviour in livestock but not for farrowing prediction [35,36].

## 5. Conclusions

In this study, ear tag acceleration data was modeled to provide first- and second-stage alarms before the onset of farrowing. A “first-stage” alarm was related to increased activity of sows when nest-building behaviour started. A “second-stage” alarm was generated when the trend in sow activity changed due to the end of nest-building behaviour. In the validation dataset, the median time of a “first-stage” alarm was 9 h 38 min before the onset of farrowing, while the median time of the “second-stage” alarm was 2 h 4 min before the onset of farrowing. Implementation of the developed model in a monitoring system of pens with the possibility of temporary crating and practical application of the tool on farms could help to achieve a compromise between the needs of the farmer, the sow and her piglets. A “first-stage” alarm could be particularly useful to indicate when a farrowing pen should be prepared for the impending farrowing. A “second-stage” alarm could indicate that most nest-building behaviour is finished and sows can be confined in crates.

## Figures and Tables

**Table 1:** Dataset divided into training and validation.

Pen type	Training	Validation
SWAP	9	9
Trapezoid	9	9
Wing	9	8
Total	27	26

**Table 2:** Training dataset, duration between onset of farrowing and time of alarms and duration from the “second-stage” alarm to the end of farrowing.

Sow Id	“First-Stage” Alarm	“Second-Stage” Alarm	Duration from “Second-Stage” Alarm to End of Farrowing
147002 7	No alarm	No alarm	No alarm
147003 8	07:06:01	02:21:01	08:10:01
147030 9	−46:45:59	−50:00:59	−44:11:59
147031 9	06:00:01	−00:29:59	01:33:01
147038 9	No alarm	No alarm	No alarm
147042 9	14:37:01	−02:22:59	01:12:01
147044 18	No alarm	No alarm	No alarm
147045 9	08:08:01	04:53:01	07:53:01
147071 8	04:52:01	−00:22:59	10:14:01
147072 8	00:28:01	−17:31:59	−13:42:59
147073 8	05:21:01	−03:38:59	−01:27:59
147106 2	08:25:01	02:40:01	06:23:01
147127 10	05:49:01	−07:10:59	−01:32:59
147142 10	No alarm	No alarm	No alarm
147142 2	No alarm	No alarm	No alarm
147143 20	00:38:01	−02:36:59	00:15:01
147146 9	08:04:01	03:49:01	05:21:01
147149 9	59:47:01	54:47:01	58:23:01
147150 9	06:55:01	03:25:01	06:03:01
147156 2	08:35:01	05:05:01	06:57:01
147158 2	13:33:01	06:48:01	12:38:01
147172 16	66:02:01	59:47:01	62:41:01
147192 8	06:17:02	−00:12:58	01:41:02
147193 23	09:46:01	00:31:01	05:54:01
147310 21	15:01:01	07:46:01	09:09:01
147317 23	05:44:01	00:44:01	03:32:01
147323 18	10:12:01	03:57:01	07:58:01

Negative duration means that either a first- or second-stage alarm was generated after the onset of farrowing, or a “second-stage” alarm was generated after the end of farrowing.

**Table 3:** Validation dataset, duration between onset of farrowing and time of alarms, and duration from a “second-stage” alarm to the end of farrowing.

Sow Id	“First-Stage” Alarm	“Second-Stage” Alarm	Duration from “Second-Stage” Alarm to end of Farrowing
147001 8	05:56:01	−00:33:59	02:56:01
147002 16	10:26:01	−03:03:59	−02:04:59
147026 9	06:33:01	−01:11:59	02:49:01
147037 18	No alarm	No alarm	No alarm
147041 9	06:03:01	02:03:00	07:00:01
147044 9	No alarm	No alarm	No alarm
147079 8	No alarm	No alarm	No alarm
147082 8	08:30:00	05:15:01	08:35:01
147121 9	13:18:01	06:48:01	08:44:01
147127 2	25:05:01	19:50:01	26:13:01
147140 20	03:42:30	00:12:30	02:12:30
147144 2	01:58:01	−02:01:58	01:33:01
147145 2	11:02:01	02:47:01	08:58:01
147150 14	08:51:27	02:06:27	09:11:27
147154 19	19:17:01	01:32:01	05:55:01
147156 10	07:32:01	03:32:01	07:27:01
147159 2	No alarm	No alarm	No alarm
147188 5	No alarm	No alarm	No alarm
147190 8	No alarm	No alarm	No alarm
147194 8	01:30:01	−03:29:59	07:30:01
147198 10	13:46:01	08:46:01	13:10:01
147222 20	No alarm	No alarm	No alarm
147310 16	No alarm	No alarm	No alarm
147347 23	20:10:01	16:55:00	22:44:01
147368 19	11:26:01	02:26:01	05:53:01
147375 19	14:46:01	02:01:01	07:01:01

Negative duration means that either first- or second-stage alarm was generated after the onset of farrowing, or “second-stage” alarm was generated after the end of farrowing.
